# Opinion: Why paediatric rheumatologists need to understand inborn errors of immunity

**DOI:** 10.3389/fped.2025.1755761

**Published:** 2026-02-05

**Authors:** Mario Abinun, Stephen Owens

**Affiliations:** 1 Translational and Clinical Research Institute, Faculty of Medical Sciences, Newcastle University, Newcastle upon Tyne, United Kingdom; 2 Paediatric Immunology, Great North Children’s Hospital, Newcastle upon Tyne Hospitals NHS Foundation Trust, Newcastle upon Tyne, United Kingdom; 3 Paediatric Immunology and Infectious Diseases, Great North Children’s Hospital, Newcastle upon Tyne Hospitals NHS Foundation Trust, Newcastle upon Tyne, United Kingdom; 4 Population Health Sciences Institute, Faculty of Medical Sciences, Newcastle University, Newcastle upon Tyne, United Kingdom

**Keywords:** autoimmunity, autoinflammatory disorders, immune dysregulatory disorders, inborn errors of immunity, primary immunodeficiency

“Rheumatology is the department of odds & ends; all the odd cases end up with us.”

John Baum, MD Professor Emeritus of Medicine and Pediatrics, University of Rochester School of Medicine and Dentistry, and a pioneer in paediatric rheumatology

## Introduction

Paediatric rheumatologists are referred a wide variety of patients affected by diseases characterised by chronic or recurrent inflammation of the musculoskeletal system and connective tissues. The “classic” rheumatological diseases include rheumatic fever, juvenile idiopathic arthritis (JIA), the spondyloarthropathies, systemic lupus erythematosus (SLE), juvenile dermatomyositis (JDM), scleroderma, and the vasculitis syndromes. However, a significant number of children present with unexplained fevers, rashes, and various musculoskeletal complaints which do not fit any defined pattern, thus “making the matter of accurate differential diagnosis extremely important” (1).

Continued paediatric rheumatology education is of utmost importance, primarily for the benefit of patients under their care (2, 3). To that effect we aim to raise the awareness of, and to bring the field and the “nomenclature” of various immunodeficiency states closer to the attention of paediatric rheumatologists.

## Lessons in history

Rheumatology, including paediatric rheumatology, was recognised as a subspecialty in the 1950's and has always been closely linked to the field of immunology, not least because rheumatic diseases are often loosely referred to as “autoimmune” conditions (1, 4). Indeed, alongside host defence against invading microorganisms, maintaining the immunologic tolerance to self-antigens is the other major function of the immune system. Preventing autoimmune tissue damage from an “overactive” immune system requires closely integrated and coordinated function of the various components of both the innate [complement system, myeloid cells, innate lymphoid cells (ILCs) - including natural killer (NK) cells] and the adaptive immune system (T and B lymphocytes), linked by numerous messenger (e.g., cytokine) pathways.

Today we understand that many of the clinical features that patients referred to paediatric rheumatology services present with, are due to underlying genetic defects of the immune system functions (5). In the initial report of the rare diseases “resulting from a failure to produce the effectors of the immune response - i.e., antibodies and sensitized lymphocytes” - termed *primary immunologic deficiency (PID),* the authors draw attention to the “associated disorders” - malignancies and autoimmunity. They stated that “*the high incidence of autoantibodies, with or without autoimmune disease, in patients with immunodeficiency has been well documented, but the reason for the association is not known*”. They speculated that it might directly relate to a genetic abnormality or to effects of the immunodeficiency, such as latent virus infection (6). Initially, autoantibodies were observed to be mainly directed to antigenic determinants of the formed elements of the blood such as red blood cells, platelets, and neutrophils, and were thought to be only rarely organ-specific (7). An “unidentified infectious agent” was suggested as a plausible explanation to the “baffling propensity” of polyarthritis occurring in some patients with agammaglobulinaemia (8), which was vividly highlighted by the *Journal of Pediatrics* in its “50 Years Ago” vignette (9). In the context of immunodeficiency, autoimmunity has been perceived not as a classical breakdown of tolerance to self-antigens but rather as tissue damage incurred as the host attempts to rid itself of foreign immunogens. Inability of the host's inherently defective immune system to completely eradicate microbial pathogens and their antigens through the usual immune pathways has been proposed to result in a compensatory, often exaggerated and protracted inflammatory response by alternative and less effective immune pathways damaging not only infected cells but also the surrounding tissues (10, 11). A seminal report confirmed that autoimmune and inflammatory manifestations frequently complicated PIDs in a large cohort of patients from the French national registry, including autoimmune cytopaenias (31%), gastrointestinal (24%), skin (14%), and rheumatic disorders (13%) (12). The authors proposed various triggers for autoimmunity and/or inflammation that included exacerbated production of type I interferon (IFN) and/or interleukin (IL)-1 production, defective negative selection of self-reactive T and B lymphocytes with high affinity, defective editing of the B-cell receptor in the periphery, defective peripheral (self)-antigen–induced cell death, gain of function (GOF) of B- or T-cell activation/effector molecules, defective regulatory T (T-reg) cells, homeostatic expansion of self-reactive T lymphocytes, an intrinsic defect in cis regulatory molecules, and defective clearance of immune complexes and apoptotic cell bodies. These can operate individually or as “a la carte” combination of misaligned mechanisms.

In addition to the well-reviewed associations between *conventional PIDs* (i.e., due to defects in complement, phagocytes, B and T lymphocytes, where affected patients manifest unusual susceptibility to various infectious agents) and rheumatic diseases (13), a novel group of *disorders of immune dysregulation* (or *primary immune regulatory disorders, PIRD*) became evident, characterized predominantly by autoimmunity, inflammation, or other immune-mediated pathology (14–16). This led the way to broadening the spectrum of inborn immune system “failures” associated with rheumatic diseases (17, 18) including the monogenic autoimmune and autoinflammatory disorders, eventually named as *inborn errors of immunity (IEI)* (19).

By the end of 1990's, the first three described *monogenic autoimmune diseases* were formally classified as PIDs - autoimmune lymphoproliferative syndrome (ALPS) caused by mutations in *CD95/FAS* gene (20), autoimmune polyendocrinopathy-candidiasis-ectodermal dystrophy (APECED) or autoimmune polyendocrinopathy syndrome (APS) type I caused by mutations in *AIRE* gene (21), and immune dysregulation, polyendocrinopathy, enteropathy, X-linked syndrome (IPEX) caused by mutations of *FOXP3* gene (22). However, as far back as 1970s, young children suffering from SLE-like disorders were reported with hereditary complement component C1q deficiency (23, 24). Resulting deficient classical complement pathway activity was postulated to underpin a failure to clear apoptotic cells leading to exaggerated inflammatory responses. This formed the basis of the “waste disposal” hypothesis to explain the pathogenesis of SLE (25, 26).

In parallel, an entirely novel group of diseases related to *periodic fever syndromes* initially described in 1950s (27, 28) was added to the canon, heralded by the finding of *MEFV* gene mutations as a cause of familial Mediterranean fever (FMF) (29–31) and of *TNFR1* gene mutations defining a family of dominantly inherited autoinflammatory syndromes (32).The ever-expanding spectrum of the *monogenic autoinflammatory diseases* refers to the inborn disorders of the innate immune system, with presentations characterized by episodes of systemic inflammation that are mediated largely by myeloid cells and by lack of autoantibodies and/or autoreactive T lymphocytes, thus distinguishing them from autoimmune diseases (33–35). Fascinatingly, C1q deficiency is currently understood and classified as an interferonopathy, one of the subcategories of autoinflammatory diseases (36).

## Semantics and nomenclature

The term “*primary immunologic deficiency (PID)*”, as per the first report by the World Health Organisation (WHO) Committee on classification of PIDs referred to a group of diseases caused by a failure of either B lymphocyte/humoral immunity and/or of thymus-dependent T lymphocyte immunity, where affected individuals presented with markedly increased susceptibility to various infectious agents manifesting usually (but not exclusively) in early childhood (6). Although primary complement and phagocytic cell defects were not initially included, over the next 5 decades the term PID expanded to comprise disorders resulting from mutations in an ever-increasing number of genes involved in immune host defence (both of innate and adaptive immunity) and/or immunoregulation (central/thymic and peripheral tolerance) as evidenced by numerous WHO and later International Union of Immunological Societies (IUIS) Committees updates. However, as stated in the 2017 update “this terminology does limit the conceptualization of disorders to those in which susceptibility to infection is the main manifestation. The improving recognition of immune dysregulation diseases, including the growing field of autoinflammatory disorders and interferonopathies, has mandated that a more encompassing terminology be used”, i.e., IEI (19).

Changes to accepted nomenclature are continuously being debated (37–41) confirming the astute prediction published at the dawn of PIDs coming into existence (42) by one of the pioneers in the field: “Like all hypothetical schemes, based on incomplete knowledge and imperfect interpretation, it must be considered tentative and useful mainly in establishing hypotheses to be demolished by future investigation” (43).

The latest update in the IUIS Committee classification lists 508 different genes known to cause IEIs that can present clinically with various manifestations, from increased susceptibility to infections, autoimmunity, autoinflammation, allergy, bone marrow failure, and/or malignancy (44). Importantly for understanding the underlying pathophysiology leading to the phenotypic (clinical) manifestations of these disorders, immunodeficiency (e.g., susceptibility to viral infections) and immune dysregulation caused by different mutation(s) of the same gene (e.g., *STAT2*) often overlap (45). Closer to the everyday paediatric rheumatology practice, this dichotomy is well illustrated by IL-1 mediated disorders that may present as bacterial infections if the downstream signalling pathway (IRAK4/MyD88) is non-functional (i.e., deficiency) or as cryopyrin-associated periodic fever syndrome (CAPS) due to overproduction of IL-1 in GOF mutations in *NLPR3* gene (46, 47). Drawing from this knowledge into the modern era management, it should be expected that biologic therapies targeting particular (specific) immunologically/pro-inflammatory active molecules and/or pathways are capable of mimicking the naturally occurring IEIs (often deficiency, but occasionally pro-inflammatory) and causing potentially serious side effects (48–51).

## Discussion – or what has it all to do with paediatric rheumatology?

It is within this maze of presentations and overlapping phenotypes that paediatric rheumatologists of the day must navigate, explore and eventually succeed in assessing and diagnosing the patient correctly (5, 52). Often, the problem is a “novel” disorder (although most likely unknowingly encountered previously), eluding the traditional evidence-based approach to clinical decision-making (53). One memorable patient of ours strikingly illustrates the evolving pattern and myriad variations in clinical presentations which characterise many of these disorders, as well as the anguish of the patient, parents, and the medical team while “searching for the unknown” (Table 1).

**Table 1:** The “open book of medicine”: timeline to diagnosis, with evolving & various features over 14 years [patient No 6; table S5 and figure S5-A,B; Zhou et al. NEJM 2014;370:911–20 (54)].

Age	Features/Dg	Investigations	Treatment	Complications
6 months	Recurrent (2–6 weeks), transient asymptomatic crops of erythematous dermal nodules/plaques	Skin bx - NLCH/GEH		
18 months	Recurrent, transient stroke episodes	Brain MRI – thalamic infarct	Aspirin (transient.)	
		Extensive investing. (all N)		
4 years	Evans Sy.	DCT + ve	Steroids	
		Extensive immunological	Rituximab	
		Investigations (all N)		
6–8 years	Hypersplenism	Liver bx - HNRH	Splenectomy	
	Portal hypertension		Spleno-renal shunt	Arterial Hypertension
	Oesophageal varices		HSCT discussed	IVIg replacement
	? complex MSAID			
9–10 years	Hepato-pulmonary Sy.		Orthoptic Liver transplant	Shunt patency problems/TIPSS
	? Immune dysregulatory disease			Liver VOD
	? Pro-thrombotic (hypercoagulability) state			IVC obstruction/Pleural effusions
	? Endothelial disorder			Anaemia/CMV reactivation
				Heart failure/RC
12–13 years	Relapse Hepato-pulmonary Sy.		? Palliation/2nd OLT/HSCT	
14 years	ADA2 deficiency (novel disease)			Died

NLCH, non-Langerhans's cell histiocytosis; GEH, generalised eruptive histiocytoma; MRI, magnetic resonance imaging; N, within normal; DCT, direct Coombs test; HNRH, hepatic nodular regenerative hyperplasia; MSAID, multi-system autoimmune disease; HSCT, haematopoietic stem cell transplantation; TIPPS, Transjugular Intrahepatic Portosystemic Shunt; VOD, veno-occlusive disease; IVC, Inferior vena cava; CMV, cytomegalovirus; RC, restrictive cardiomyopathy; ADA2, adenosine deaminase 2; OLT, orthoptic liver transplant.

Speciality teams involved (Immunology leading): - Neurology, Dermatology, Rheumatology, Hepatology, Haematology, Respiratory, Nephrology, Cardiology, Intensive Care, Palliative Care - Leeds Paediatric Liver Transplantation team (OLT).

Secondary opinions: - Professor B. Ziegler, Department of Dermatology, University of Innsbruck, Austria (skin biopsy) - GOSH/London - Immunology, Cardiology, Respiratory, Rheumatology/Vasculitis/Immunology, Liver/Kings College Hospital (2nd opinion) - Birmingham Paediatric Liver Transplantation team (opinion re 2nd OLT).

The unprecedented advances in development of modern genetic analysis allowed for an ever-increasing number of novel monogenic disease entities to be defined in the last two decades and there is no reason to doubt that this is to continue (55, 56). The fact that almost half of the newly reported genes underlying IEIs in the previous two years refer to either autoinflammatory or immune dysregulation disorders is a strong proof that the field of IEIs is inseparably intertwined with rheumatology (5, 44).

Therefore, the main practice implications for paediatric rheumatologists are as follows:

The most important clinical acumen is to discern the “*red flags*” which may signal the possibility of an underlying monogenic IEI as a cause for rheumatological presentation. Broadly, these are features that are unusual, atypical (in pattern, severity, age of onset) and evolving in multisystem involvement often requiring very specific “precision and personalised” therapies (Box 1).

Box 1 **"Red Flags" for suspecting a monogenic IEI paediatric rheumatology. Adapted from (85).**

○ (Very) early onset with a severe course - unusual
○ Constellation of multiple autoimmune disorder - unusual
○ Evolution of autoimmunity with accrual of different organ system involvement (multisystem involvement) - a pattern
○ Family history (similar disorder; sex-linked/male-only) and parental consanguinity (autosomal recessive trait) - a pattern
○ Predisposition to infection (usually of early onset, frequent and severe) - unusual (pattern)

The modern-day paediatric rheumatologist then requires ready access to *current diagnostic tools*, including functional immunology tests, cytokine profiling, interferon-stimulated gene (ISG) expression, and whole genome sequencing (WGS). These are powerful tools in the diagnosis and management of rheumatological conditions (informing “precision treatments”) although not without caveats (e.g., the accurate evaluation and interpretation of results) of which practicing paediatric rheumatologists ought to be well aware (57–62).

They benefit from *collaborative working* with their colleagues in paediatric immunology and from managed clinical networks through which experience and learning gleaned from the care of small numbers of children with very rare conditions can be shared more widely. In this way, the ongoing dialogue between immunology and rheumatology continues to enrich both specialities and drives improvements in the care of their patients. Many patients under our care benefited from multidisciplinary team (MDT) approach via combined paediatric rheumatology and immunology clinics (initially set up as “periodic fever clinics”, later combined with other specialities), as well as by national (63–66) and international collaboration (67–71). Our team contributed to identification of several novel IEI disorders with significant “rheumatological” phenotypes that were part of their clinical presentation (72–78). These are rare diseases, often with no more than a single or handful of patients initially observed by astute clinicians; yet in the following years it transpired that some (e.g., deficiency of adenosine deaminase 2, DADA2) are among the most common monogenic immunodysregulatory and/or autoinflammatory diseases (79).

Today's options for “*personalised*” *treatments* are unparalleled in comparison even to those of yesterday, let alone to the historical “therapeutic pyramid” approach (80–82). However, as some patients with monogenic immune dysregulation and/or autoinflammatory disorders are resistant even to the very specific and targeted treatments, there is a valid role for haematopoietic stem cell transplantation (HSCT) in their management (83) and prospect for gene therapy and/or editing in the near future are promising (84).

## Summary

Advances in immunology over the last 30 years have revolutionised the field of rheumatology. Today many of the clinical phenotypes presenting to rheumatology clinics have clearly defined genetic aetiologies through which disruptions in carefully regulated immune pathways lead to autoimmunity, autoinflammation and other immune-mediated pathology, which are understood with ever more clarity. Dozens of newly-recognised primary immune regulatory disorders have taken their place alongside the classical PIDs of old, in a list of more than 500 monogenic inborn errors of immunity, as described in the latest IUIS Classification (44, 56).

## References

[1] Shaller JG. The history of pediatric rheumatology. Pediatr Res. (2005) 58:997–1007. 10.1203/01.PDR.0000182823.85717.48 16183803

[2] McColl J Mwizerwa O Scott C Tse SML Foster HE. Pediatric rheumatology education: the virtual frontier a review. Pediatr Rheumatol. (2024) 22:60. 10.1186/s12969-024-00978-0 PMC11155138 38840147

[3] Turnier JL Canna SW. Insights from the 2024 pediatric rheumatology basic/translational years in review. Pediatr Rheumatol. (2025) 23:57. 10.1186/s12969-025-01102-6 PMC12100868 40410775

[4] Sogkas G Witte T. The link between rheumatic disorders and inborn errors of immunity. eBioMedicine. (2023) 90:104501. 10.1016/j.ebiom.2023.104501 36870198 PMC9996386

[5] Tobin JM Cooper MA. Rheumatologic and autoimmune features of inborn errors of immunity: implications for diagnosis and management. J Hum Immun. (2025) 1(3):e20250034. 10.70962/jhi.20250034 40842819 PMC12365696

[6] Fudenberg HH Good RA Hitzig WH Kunkel HG Roitt IM Rosen FS Classification of the primary immune deficiencies: WHO recommendation. N Engl J Med. (1970) 283(12):656–7 (idem: Bullet WHO. 1971;45(1):125-42; Pediatrics. 1971;47(5):927-46) (these are “parallel” publications). 10.1056/NEJM197009172831211 5456822

[7] Rosen FS. Autoimmunity and immunodeficiency disease. Ciba Found Symp. (1987) 129:135–48. 3315500 10.1002/9780470513484.ch10

[8] McLaughlin JF Schaller J Wedgwood RJ. Arthritis and immunodeficiency. J Pediatr. (1972) 81:801–3. 10.1016/S0022-3476(72)80110-0 5074359

[9] Ashkenazi L Hashkes PJ. 50 Years ago in the journal of pediatrics: immunodeficiency and autoimmunity—are there common pathways? J Pediatr. (2022) 249:58. 10.1016/j.jpeds.2022.07.022 36216479

[10] Arkwright PD Abinun M Cant AJ. Autoimmunity in human primary immunodeficiency diseases. Blood. (2022) 99(8):2694–702. 10.1182/blood.V99.8.2694 11929755

[11] Etzioni A. Immune deficiency and autoimmunity. Autoimm Rev. (2003) 2:364–9. 10.1016/S1568-9972(03)00052-1 14550878

[12] Fischer A Provot J Jais JP Alcais A Mahlaoui N. (Members of the CEREDIH French PID study group). autoimmune and inflammatory manifestations occur frequently in patients with primary immunodeficiency. J Allergy Clin Immunol. (2017) 140:1388–93.e8. 10.1016/j.jaci.2016.12.978 28192146

[13] Torgerson TR. Immunodeficiency diseases with rheumatic manifestations. Pediatr Clin N Am. (2012) 59:493–507. 10.1016/j.pcl.2012.03.010 22560581

[14] Notarangelo LD Casanova JL Fischer A Puck JM Rosen FS Seger R Primary immunodeficiency diseases: an update. J Allergy Clin Immunol. (2004) 114:677–87. 10.1016/j.jaci.2004.06.044 15356576

[15] Bonilla FA Khan DA Ballas ZK Chinen J Frank MM Hsu JT Practice parameter for the diagnosis and management of primary immunodeficiency. J Allergy Clin Immunol. (2015) 136:1186–205. 10.1016/j.jaci.2015.04.049 26371839

[16] Notarangelo LD Bachetta R Casanova J-L Su HC. Human inborn errors of immunity: an expanding universe. Sci Immunol. (2020) 5:eabb1662. 10.1126/sciimmunol.abb1662 32651211 PMC7647049

[17] Dimitriades VR Sorensen R. Rheumatologic manifestations of primary immunodeficiency diseases. Clin Rheumatol. (2016) 35:843–50. 10.1007/s10067-016-3229-6 26971790

[18] Allenspach E Torgerson TR. Autoimmunity and primary immunodeficiency disorders. J Clin Immunol. (2016) 36(Suppl 1):S57–67. 10.1007/s10875-016-0294-1 27210535

[19] Picard C Gaspar HB Al-Herz W Bousfiha A Casanova JL Chatila T International union of immunological societies: 2017 primary immunodeficiency diseases committee report on inborn errors of immunity. J Clin Immunol. (2018) 38(1):96–128. 10.1007/s10875-017-0464-9 29226302 PMC5742601

[20] Rieux-Laucat F Le Deist F Hivroz C Roberts IA Debatin KM Fischer A Mutations in fas associated with human lymphoproliferative syndrome and autoimmunity. Science. (1995) 268(5215):1347–9. 10.1126/science.7539157 7539157

[21] Finnish-German APECED Consortium. An autoimmune disease, APECED, caused by mutations in a novel gene featuring two PHD-type zinc-finger domains. Nat Genet. (1997) 17(4):399–403. 10.1038/ng1297-399 9398840

[22] Bennett CL Christie J Ramsdell F Brunkow ME Ferguson PJ Whitesell L The immune dysregulation, polyendocrinopathy, enteropathy, X-linked syndrome (IPEX) is caused by mutations of FOXP3. Nat Genet. (2001) 27(1):20–1. 10.1038/83713 11137993

[23] Berkel AI Loos M Sanal O Mauff G Gungen Y Ors U Clinical and immunological studies in a case of selective complete C1q deficiency. Clin Exp Immunol. (1979) 38:52–63. 527255 PMC1537840

[24] Mikuska M Stefanovic Z Miletic V. Systemic lupus erythematosus-like disease in two siblings with complete C1q deficiency. Period Biol. (1983) 85(Suppl 3):271–3.

[25] Schifferli JA Ng YC Pacaud J-P Walport MJ. The role of hypocomplementaemia and low erythrocyte complement receptor type 1 numbers in determining abnormal immune complex clearance in humans. Clin Exp Immunol. (1989) 75:329–35. 2522842 PMC1541944

[26] Walport MJ. Complement. N Engl J Med. (2001) 344(14):1058–66 (part 1); idem (15):1140-44 (part 2). 10.1056/NEJM200104053441406 11287977

[27] Reimann HA. Periodic disease; a probable syndrome including periodic fever, benign paroxysmal peritonitis, cyclic neutropenia and intermittent arthralgia. J Am Med Assoc. (1948) 136(4):239–44. 10.1001/jama.1948.02890210023004 18920089

[28] Reimann HA. Periodicity in disease. New Engl J Med. (1957) 256(14):652–3. 10.1056/NEJM195704042561407 13451912

[29] The International FMF Consortium. Ancient missense mutations in a new member of the RoRet gene family are likely to cause familial Mediterranean fever. Cell. (1997) 90(4):797–807. 10.1016/S0092-8674(00)80539-5 9288758

[30] French FMF Consortium. A candidate gene for familial Mediterranean fever. Nat Genet. (1997) 17(1):25–31. 10.1038/ng0997-25 9288094

[31] Samuels J Aksentijevich I Torosyan Y Centola M Deng Z Mansfield E Familial Mediterranean fever at the millennium. Clinical spectrum, ancient mutations, and a survey of 100 American referrals to the national institutes of health. Medicine (Baltimore). (1998) 77(4):268–97. 10.1097/00005792-199807000-00005 9715731

[32] McDermott MF Aksentijevich I Galon J Germline mutations in the extracellular domains of the 55 kDa TNF receptor, TNFR1, define a family of dominantly inherited autoinflammatory syndromes. Cell. (1999) 97(1):133–44. 10.1016/S0092-8674(00)80721-7 10199409

[33] Stoffels M Kastner DL. Old dogs, new tricks: monogenic autoinflammatory disease unleashed. Annu Rev Genomics Hum Genet. (2016) 17:245–72. 10.1146/annurev-genom-090413-025334 27362340

[34] Moghaddas F Masters SL. The classification, genetic diagnosis and modelling of monogenic autoinflammatory disorders. Clin Sci. (2018) 132:1901–24. 10.1042/CS20171498 PMC6123071 30185613

[35] Meertens B Hoste L Tavernier SJ Haerynck F. The riddle of recurrent fever: a clinical approach to pediatric autoinflammatory diseases. Front Pediatr. (2024) 12:1448176. 10.3389/fped.2024.1448176 39618694 PMC11605516

[36] Triaille C Rao NM Rice GI Seabra L Sutherland FJH Bondet V Hereditary C1q deficiency is associated with type 1 interferon-pathway activation and a high risk of central nervous system inflammation. J Clin Immunol. (2024) 44(8):185. 10.1007/s10875-024-01788-5 39196411 PMC11358312

[37] Al-Herz W Notarangelo LD. Classification of primary immunodeficiency disorders: one-fits-all does not help anymore. Clin Immunol. (2012) 144:24–5. 10.1016/j.clim.2012.05.003 22659031

[38] Akalu YT Bogunovic D. Inborn errors of immunity: an expanding universe of disease and genetic architecture. Nat Rev Genet. (2024) 25(3):184–95. 10.1038/s41576-023-00656-z 37863939 PMC13489759

[39] Seidel MG. Rethinking PIDs: why the distinction between primary and secondary immune disorders is more frequently relevant than that between inborn and acquired errors of immunity. J Allergy Clin Immunol. (2024) 153(6):1543–5. 10.1016/j.jaci.2024.01.018 38316271

[40] Turvey SE Biggs CM James EL Hildebrand KJ. Should “primary immune disorder” replace “inborn error of immunity"? names matter, but there is room for both. J Allergy Clin Immunol. (2024) 153(6):1546–7. 10.1016/j.jaci.2024.04.007 38649118

[41] Henrickson SE. Evolution of the concept of immune dysregulation and current classification. J Allergy Clin Immunol. (2025) 155:89–91. 10.1016/j.jaci.2024.10.010 39428080

[42] Geha RS. Charles A. Janeway and Fred S. Rosen: the discovery of gamma globulin therapy and primary immunodeficiency diseases at Boston children’s hospital. J Allergy Clin Immunol. (2005) 116(4):937–40. 10.1016/j.jaci.2005.07.025 16229111

[43] Janeway CA. Progress in immunology. Syndromes of diminished resistance to infection. J Pediatr. (1968) 72(6):885–903. 10.1016/s0022-3476(68)80446-9 4173567

[44] Poli MC Aksentijevich I Bousfiha AA Cunningham-Rundles C Hambleton S Klein C Human inborn errors of immunity: 2024 update on the classification from the international union of immunological societies expert committee. J Hum Immun. (2025) 1(1):e20250003. 10.70962/jhi.20250003 41608114 PMC12829761

[45] Duncan CJA Hambleton S. Human disease phenotypes associated with loss and gain of function mutations in STAT2: viral susceptibility and type I interferonopathy. J Clin Immunol. (2021) 41(7):1446–56. 10.1007/s10875-021-01118-z 34448086 PMC8390117

[46] Picard C von Bernuth H Ghandil P Chrabieh M Levy O Arkwright PD Clinical features and outcome of patients with IRAK-4 and MyD88 deficiency. Medicine (Baltimore). (2010) 89(6):403–25. 10.1097/MD.0b013e3181fd8ec3 21057262 PMC3103888

[47] Henderson C Goldbach-Mansky R. Monogenic IL-1 mediated autoinflammatory and immunodeficiency syndromes: finding the right balance in response to danger signals. Clin Immunol. (2010) 135:210–22. 10.1016/j.clim.2010.02.013 20353899 PMC2856775

[48] Maródi L Casanova JL. Primary immunodeficiencies may reveal potential infectious diseases associated with immune-targeting mAb treatments. J Allergy Clin Immunol. (2010) 126(5):910–7. 10.1016/j.jaci.2010.08.009 20943260

[49] Labrosse R Haddad E. Immunodeficiency secondary to biologics. J Allergy Clin Immunol. (2023) 151:686–90. 10.1016/j.jaci.2023.01.012 36706964

[50] Suntharalingam G Perry MR Ward S Brett SJ Castello-Cortes A Brunner MD Cytokine storm in a phase 1 trial of the anti-CD28 monoclonal antibody TGN1412. N Engl J Med. (2006) 355:1018–28. 10.1056/NEJMoa063842 16908486

[51] Abinun M. Foreword. In: Cron RQ Behrens EM, editors. Cytokine Storm Syndrome. 2nd ed. Cham: Springer (2024). p. x–xii. Adv Exp Medicine Biology, vol. 1448.

[52] Abinun M Torgerson TR Inborn errors of immunity and rheumatic disease (chapter 49). In: Petty RE, editor. Textbook of Pediatric Rheumatology. 9th ed. Philadelphia: Elsevier Inc. (in press).

[53] Isaacs D Fitzgerald D. Seven alternatives to evidence based medicine. Br Med J. (1999) 319:1618. 10.1136/bmj.319.7225.1618 10600968 PMC28313

[54] Zhou Q Yang D Ombrello AK Zavialov AV Toro C Stone DL Early-onset stroke and vasculopathy associated with mutations in ADA2. N Engl J Med. (2014) 370(10):911–20. 10.1056/NEJMoa1307361 24552284 PMC4193683

[55] do Nascimento RRNR Quaio CRD'AC Chung CH de Moraes Vasconcelos D Sztajnbok FR Rosa Neto NS Principles of clinical genetics for rheumatologists: clinical indications and interpretation of broad-based genetic testing. Adv Rheumatol. (2024) 64:59. 10.1186/s42358-024-00400-z 39143637 PMC13488838

[56] Kindle G Alligon M Albert MH Buckland M Edgar JD Gathmann B Inborn errors of immunity: manifestation, treatment, and outcome—an ESID registry 1994–2024 report on 30,628 patients. J Hum Immun. (2025) 1(3):e20250007. 10.70962/jhi.20250007 41347188 PMC12674179

[57] Schindewolf E Kilich G Westerfer D Vandergrift D Rajagopalan R Sullivan KE. Mastering genetics for your clinic: a step by step guide. J Allergy Clin Immunol. (2025) 156(5):1125–1132. 10.1016/j.jaci.2025.07.027 40789456 PMC13157676

[58] Sullivan KE. The scary world of variants of uncertain significance (VUS): a hitchhiker’s guide to interpretation. J Allergy Clin Immunol. (2021) 147:492–4. 10.1016/j.jaci.2020.06.011 32598897

[59] Gallo PM Kim J McNerney KO Diorio C Foley C Kagami L Serum cytokine panels in pediatric clinical practice. J Allergy Clin Immunol. (2025) 155(2):594–604.e5. 10.1016/j.jaci.2024.08.030 39303891 PMC12502881

[60] Knight V Sepiashvili L. Cytokine testing and challenges for diagnostic and clinical monitoring use. J Allergy Clin Immunol. (2025) 155:410–3. 10.1016/j.jaci.2024.11.025 39613111

[61] Triaille C Touzot F. Cytokine testing: bench-ready, bedside pending. J Allergy Clin Immunol. (2025) 156(1):200. 10.1016/j.jaci.2025.03.027 40249355

[62] Gallo PM. Cytokine testing: asking the right questions. J Allergy Clin Immunol. (2025) 156(6):1776–7. 10.1016/j.jaci.2025.08.023 41020747

[63] Ramesh V Bernardi B Stafa A Garone C Franzoni E Abinun M Intracerebral large artery disease in Aicardi-Goutières syndrome implicates SAMHD1 in vascular homeostasis. Dev Med Child Neurol. (2010) 52(8):725–32. 10.1111/j.1469-8749.2010.03727.x 20653736

[64] McCann LJ McPartland J Barge D Strain L Bourn D Calonje E Phenotypic variations of cartilage hair hypoplasia: granulomatous skin inflammation and severe T cell immunodeficiency as initial clinical presentation in otherwise well child with short stature. J Clin Immunol. (2014) 34(1):42–8. 10.1007/s10875-013-9962-6 24217815 PMC7086599

[65] Duncan CJA Dinnigan E Theobald R Grainger A Skelton AJ Hussain R Early-onset autoimmune disease due to a heterozygous loss-of-function mutation in TNFAIP3 (A20). Ann Rheum Dis. (2018) 77:783–6. 10.1136/annrheumdis-2016-210944 28659290 PMC5909743

[66] Altmann T Torvell M Owens S Mitra D Sheerin NS Morgan BP Complement factor I deficiency: a potentially treatable cause of fulminant cerebral inflammation. Neurol Neuroimmunol Neuroinflamm. (2020) 7(3):e689. 10.1212/NXI.0000000000000689 32098865 PMC7051217

[67] Rensing-Ehl A Warnatz K Fuchs S Schlesier M Salzer U Draeger R Clinical and immunological overlap between autoimmune lymphoproliferative syndrome and common variable immunodeficiency. Clin Immunol. (2010) 137(3):357–65. 10.1016/j.clim.2010.08.008 20832369

[68] Leiding JW Vogel TP Santarlas VGJ Mhaskar R Smith MR Carisey A Monogenic early-onset lymphoproliferation and autoimmunity: natural history of STAT3 gain-of-function syndrome. J Allergy Clin Immunol. (2023) 151(4):1081–95. 10.1016/j.jaci.2022.09.002 36228738 PMC10081938

[69] Maccari ME Fuchs S Kury P Andrieux G Völkl S Bengsch B A distinct CD38+CD45RA+ population of CD4+, CD8+, and double-negative T cells is controlled by FAS. J Exp Med. (2021) 218(2):e20192191. 10.1084/jem.20192191 33170215 PMC7658692

[70] Hägele P Staus P Scheible R Uhlmann A Heeg M Klemann C (ALPID study group). diagnostic evaluation of paediatric autoimmune lymphoproliferative immunodeficiencies (ALPID): a prospective cohort study. Lancet Haematol. (2024) 11(2):e114–26. 10.1016/S2352-3026(23)00362-9 38302222

[71] Buso H Triaille C Flinn AM Gennery AR. Update on hereditary C1q deficiency: pathophysiology, clinical presentation, genotype and management. Curr Opin Allergy Clin Immunol. (2024) 24(6):427–33. 10.1097/ACI.0000000000001034 39479952

[72] Angulo I Vadas O Garçon F Banham-Hall E Plagnol V Leahy TR Phosphoinositide 3-kinase *δ* gene mutation predisposes to respiratory infection and airway damage. Science. (2013) 342(6160):866–71. 10.1126/science.1243292 24136356 PMC3930011

[73] Holzinger D Fassl SK de Jager W Lohse P Röhrig UF Gattorno M Single amino acid charge switch defines clinically distinct proline-serine-threonine phosphatase-interacting protein 1 (PSTPIP1)-associated inflammatory diseases. J Allergy Clin Immunol. (2015) 136(5):1337–45. 10.1016/j.jaci.2015.04.016 26025129 PMC6591125

[74] Zhou Q Yu X Demirkaya E Deuitch N Stone D Tsai WL Biallelic hypomorphic mutations in a linear deubiquitinase define otulipenia, an early-onset autoinflammatory disease. Proc Natl Acad Sci U S A. (2016) 113(36):10127–32. 10.1073/pnas.1612594113 27559085 PMC5018768

[75] Cuchet-Lourenço D Eletto D Wu C Plagnol V Papapietro O Curtis J Biallelic RIPK1 mutations in humans cause severe immunodeficiency, arthritis, and intestinal inflammation. Science. (2018) 361(6404):810–3. 10.1126/science.aar2641 30026316 PMC6529353

[76] Wang L Aschenbrenner D Zeng Z Cao X Mayr D Mehta M Gain-of-function variants in SYK cause immune dysregulation and systemic inflammation in humans and mice. Nat Genet. (2021) 53(4):500–10. 10.1038/s41588-021-00803-4 33782605 PMC8245161

[77] Stremenova Spegarova J Sinnappurajar P Al Julandani D Navickas R Griffin H Ahuja M A *de novo* TLR7 gain-of-function mutation causing severe monogenic lupus in an infant. J Clin Investig. (2024) 134(13):e179193. 10.1172/JCI179193 38753439 PMC11213501

[78] van der Made CI Kersten S Chorin O Engelhardt KR Ramakrishnan G Griffin H Expanding the PRAAS spectrum: *de novo* mutations of immunoproteasome subunit β-type 10 in six infants with SCID-Omenn syndrome. Am J Hum Genet. (2024) 111(4):791–804. 10.1016/j.ajhg.2024.02.013 38503300 PMC11023912

[79] Lee PY Davidson BA Abraham RS Alter B Arostegui JI Bell K Evaluation and management of deficiency of adenosine deaminase 2: an international consensus statement. JAMA Netw Open. (2023) 6(5):e2315894. 10.1001/jamanetworkopen.2023.15894 37256629

[80] Shishov M Weiss PF Levy DM Chang JC Angeles-Han ST Ogbu EA (Pediatric rheumatology collaborative study group advisory council). 25 years of biologics for the treatment of pediatric rheumatic disease: advances in prognosis and ongoing challenges. Arthritis Care Res (Hoboken). (2025) 77(5):573–83. 10.1002/acr.25482 39651592 PMC12915560

[81] Dinarello CA. Anti-inflammatory agents: present and future. Cell. (2010) 140(6):935–50. 10.1016/j.cell.2010.02.043 20303881 PMC3752337

[82] Levinson JE Wallace CA. Dismantling the pyramid. J Rheumatol Suppl. (1992) 33:6–10. 1593604

[83] Abinun M Slatter MA. Haematopoietic stem cell transplantation in paediatric rheumatic disease. Curr Opin Rheumatol. (2021) 33:387–97. 10.1097/BOR.0000000000000823 34261117

[84] Rigamonti C Bulté D Barzaghi F Mesa-Nuñez C Basso-Ricci L Pettinato E Gene therapy supports long-term reconstitution of patient hematopoietic stem cells in deficiency of adenosine deaminase 2. Mol Ther Methods Clin Dev. (2025) 33(4):101624. 10.1016/j.omtm.2025.101624 41321395 PMC12662005

[85] Abinun M Sullivan K. Primary immunodeficiencies and rheumatic diseases (chapter 43). In: Petty RE, editor. Textbook of Pediatric Rheumatology. 8th ed. Philadelphia: Elsevier Inc. (2021). p. 575–85.

